# Psychosocial Impact of Breast Density Notification Through Breast Cancer Screening: A Qualitative Interview Study

**DOI:** 10.1111/hex.70539

**Published:** 2026-03-09

**Authors:** Emma Grundtvig Gram, Claire Hudson, Nehmat Houssami, Kirsten McCaffery, Jennifer Isautier, Brooke Nickel

**Affiliations:** ^1^ Sydney Health Literacy Lab, School of Public Health, Faculty of Medicine and Health The University of Sydney Sydney New South Wales Australia; ^2^ Center for General Practice, Department of Public Health University of Copenhagen Copenhagen Denmark; ^3^ Wiser Healthcare, School of Public Health The University of Sydney Sydney New South Wales Australia; ^4^ The Daffodil Centre, A Joint Venture With Cancer Council NSW The University of Sydney Sydney New South Wales Australia

**Keywords:** breast cancer, breast cancer screening, breast density, interviews, psychosocial, qualitative methods, women's health

## Abstract

**Introduction:**

Breast density notification is now implemented in the United States and parts of Australia and Canada despite limited understanding of how it impacts women and their psychosocial health. This study explored the psychosocial impact of receiving a notification of dense breasts through a population‐based breast cancer screening programme.

**Methods:**

We interviewed 19 Australian women who indicated being anxious in a survey. This survey was part of the first randomised controlled trial aiming to quantify the psychosocial impact of breast density notifications. Semi‐structured interviews were conducted over the phone and analysed thematically to provide nuance and perspectives on the psychosocial impact.

**Results:**

The women were 42–78 years old and had diverse ethnic and educational backgrounds. Women generally perceived breast density notifications as valuable for making health decisions, citing female empowerment and the value of information in health. Women described experiences of immediate panic and worry as well as changes to their perceived individual risk of breast cancer. The breast density notification challenged women's previous perceptions of risk of breast cancer, leaving them to reinterpret their need for medical assistance. Women thus described an increased general awareness about breast cancer, which increased their likelihood of performing self‐exams and attending breast screenings.

**Discussion:**

While empowerment and increased vigilance in health might present positive strides for women's health, the derivative worry highlights the paradoxical value of health awareness and risk communication. This tension is important to consider in future notification practices and decisions on whether to implement breast density notification.

**Patient or Public Contribution:**

This study is based on interviews with 19 women who have lived experience with breast density information. The semi‐structured interview guide allowed the women to influence what was considered important and relevant.

## 1 Introduction

Breast density is defined as the ratio of fibroglandular tissue to fatty tissue in the breasts. Density increases with the proportion of fibroglandular tissues, and high density is an independent risk factor for breast cancer [1, 2, 3] and decreases the sensitivity of mammography [4]. About 30%–50% of women of screening age are estimated to have dense breasts depending on age, measurement and classification [5, 6]. Breast density notification is now implemented in the United States and regions of Australia and Canada to support personalised care and decision‐making about breast health.

However, there is limited evidence on how density notification impacts women and their psychosocial health [7, 8]. An Australian randomised controlled trial (RCT) investigates the benefits, harms and psychosocial outcomes of introducing breast density notification to one of the state‐based BreastScreen programmes [9]. Complementing the quantification of psychosocial outcomes in the trial, this study aims to explore the qualitative experience of breast density notification in women who indicated they were anxious following the receipt of their mammogram results and breast density notification. This might offer explanations of patterns in psychosocial outcomes and provide an understanding of how and why women are affected. Previous qualitative studies on breast density notifications have tended to focus on comprehensibility and preferences or have employed deductive or descriptive analytical frameworks [10, 11, 12, 13]. The Australian Government is calling for more research to understand the impact of breast density notifications to inform future practice [14].

This study explored women's experience of receiving a breast density notification as well as understanding what reporting psychosocial consequences entails. These insights could inform notification practices as well as decisions on whether to implement breast density notifications in other jurisdictions currently contemplating management of breast density [15, 16].

## 2 Materials and Methods

This qualitative study used semi‐structured interviews and thematic analysis to explore and describe the psychosocial impact of breast density notification among women who received a breast density notification and indicated anxiousness.

### 2.1 Participant Recruitment

We recruited participants via the Breast Density Notification Trial in Queensland, Australia [9, 17]. The trial population comprised women aged 40 or older with dense breasts and no abnormalities detected, attending the BreastScreen Queensland (the National screening programme). The trial included psychological outcomes such as anxiety measured by a single item in a survey (Box 1). We sampled women who had been notified of having dense breasts within the past 3 months and had indicated anxiousness (‘Agree’ or ‘Strongly Agree’) in the survey following the notification (Box 1) [9, 17]. The item was not formally validated but tested experimentally for face validity in focus groups. We sampled participants with a focus on diversity in age and educational background. We contacted participants by phone and informed them about the study, including that the interview is voluntary, does not affect screening status or care, and is confidential.

Box 1 **Anxiety measure.**

Item: ‘Please indicate the extent of your agreement or disagreement with the following statements: Receiving my screening mammogram result letter made me feel anxious’

Response items: Strongly agree, Agree, Disagree, Strongly disagree

### 2.2 Semi‐Structured Interviews

We developed the interview guide based on the literature of psychological impacts of breast cancer screening and insights from previous interviews with women in this trial about their health behaviour [17, 18, 19, 20] (Appendix 1). We hypothesised that the psychosocial impact relevant to women receiving a false‐positive result, such as impact on emotional well‐being, and worries about general health and breast health, might also apply to women experiencing anxiousness following a breast density notification [9, 18].

Interviews were semi‐structured with open‐ended questions to allow for an explorative approach and included a discussion about the general value of breast density notifications to reveal health values and encourage openness and reflection on personal experience [21]. The interview guide was used not as a checklist but as topics that could be covered depending on the individual interview. To further encourage openness, the derivative discussions were framed in third‐person abstract terms, helping participants imagine thoughts or emotions and promoting candid responses [21, 22]. For example, when respondents responded briefly, a third‐person abstract questioning technique was used to help participants imagine thoughts or emotions.

Women were interviewed over the telephone by EGG, who has a background in public health and is experienced in qualitative research on breast cancer screening. To foster deeper insights, the interviewer focused on building rapport and making space for off‐topic conversations during the interviews. For example, interviews included talking about topics not directly related to the interview, such as family life, hobbies or activities the women just came from or were about to attend. The interviewer recognised that discussing cancer and screening can be sensitive, and societal norms around personal responsibility might lead women to feel self‐conscious or ashamed if they perceive themselves as not adhering to these expectations [23]. The interviewer was mindful of these dynamics, ensuring women felt comfortable, emphasising that there is no established consensus on recommended actions after a breast density notification [24].

Nineteen interviews were conducted in September and October 2024 and lasted 13–35 min (average 24.5 min).

### 2.3 Analysis Approach

Interviews were audio‐recorded and transcribed verbatim. Recordings were treated confidentially and preserved responsibly on a secure drive. To protect participant confidentiality, the analysis is presented using pseudonyms and all identifying material was removed during transcription.

We used a thematic analysis approach providing structured identification and summarisation of patterns and themes across interviews, aiming to describe broader themes across participants' experiences [25, 26, 27]. The initial coding was done inductively by EGG, incident by incident, coding larger segments of the material to capture more general ideas or themes [27]. This was followed by double‐coding of three interviews and group discussion of codes and interpretations. After group discussion, EGG performed focused coding based on the research aim to understand what it means to indicate anxiousness and to uncover more subtle patterns across women's experiences. We used this coding strategy in order to summarise the data, identify broader themes and present common experiences rather than capturing the individual narratives, in accordance with the richness of information and personal details attainable in telephone interviews [28]. However, analysing the women's experiences, we also attended to the social context of these experiences. We continuously revisited the full transcriptions to validate and modify the analysis and interpretation.

## 3 Results

**Figure 1:**
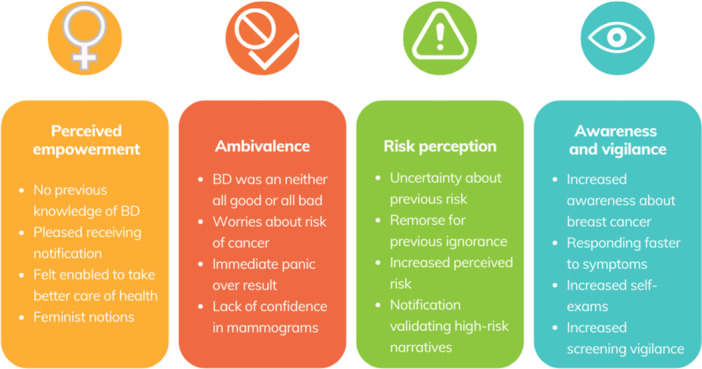
Identified themes of analysis. BD = Breast Density.

We interviewed 19 women. The women were 42–78 years old, had a diverse educational background and were primarily born in Australia, and four of them had a family history of breast cancer (Table 1). Through thematic analysis, we identified four themes: empowerment of breast density notifications, ambivalence of breast density notification, impact on risk perception, and increased awareness and vigilance in breast cancer screening and self‐examinations (Figure 1).

**Table 1:** Demographic characteristics of participants.

Characteristic	Categories	*n* (%)
Age	40s	5 (26)
	50s	7 (37)
	60s	4 (21)
	70s	3 (16)
Birth country	Asia	3 (16)
	Australia	14 (74)
	Europe	2 (10)
Educational level	University degree	10 (53)
	Diploma or certificate	4 (21)
	School or intermediate certificate	5 (26)
Family history of breast cancer	Family history	4 (21)
	No family history	15 (79)
Anxiety	Agree	9 (47)
	Strongly agree	10 (53)

### 3.1 The Empowerment of Being Notified About Breast Density

Generally, the women had not heard about breast density prior to notification: ‘*Given I had absolutely no clue, I was like: Oh, I wonder what that means. I hope that's not bad’* (Julie, 50). Based on the information they were given, they seemed to have a good understanding of what breast density was: ‘*…it puts you at a little bit higher risk*’ (Jenny, 43) and ‘*…breast cancer might be trickier to detect’* (Linda, 77).

All women stated that they were pleased with receiving the information: *‘I suppose any information is just good information to have about your health, especially with breast care: if it's something to do with my boobs, sure, I'm interested. Give me any information I can have, all of it’* (Wendy, 54). Wendy prioritised knowing as much as possible about her health, echoed by Sharon: *‘I think it's a necessary evil, really. It needs to be put out there’* (Sharon, 69). Sharon was both pleased to get the notification and considered it essential for women in general to be informed about this.

Alison similarly said that notifications were an enabler to take better care of your own health:

*‘Because you feel more informed. You are more informed. I'd rather know rather than be left in the dark and maybe having something and getting breast cancer, and they say, “Because you've got dense breasts, make sure you get checked all the time” where some women may not get regular checks. They might just forget about it or whatever’* (Alison, 69). Breast density notifications were articulated as crucial information and a source of female empowerment, enabling women to better care for their bodies as well as staying informed about their health. Such notions of empowerment related to feminism and were also expressed by the way the women talked about *‘we’* and ‘*our*’ as a group of individuals who needed better means to take care of their bodies: *‘I think we all should receive any information that's relevant to our own bodies. I think if you have the information then you can deal with it if you need to’* (Kristen, 78).

### 3.2 The Ambivalence of Breast Density Notification

Despite being contend with receiving a normal mammography screening, an accompanying notification of having dense breasts was an ambivalent experience: *‘Well, I read it and: Oh, good, there's nothing there, but when I read the density thing, I thought: Oh, goodness. I guess it sat me back a bit, and I thought: Oh, heck, should I be worried about this?’* (Janet, 62). Janet was immediately relieved that she did not have cancer, but upon reading the information that she had dense breasts, she worried that this might be bad. She further elaborated: *‘You'd like them (mammograms) to be done with confidence and to know that whatever you hear back is either, “Yes, it's all good” or “No, I need to do something” not…. It's kind of you don't know whether you need to panic or not’.* Emphasising that receiving a normal mammography screening result but also being notified about breast density was ambivalent, as the letter contained both good and bad news. The breast density notification may overshadow the good news of a normal screening result, as breast density raises issues about confidence in screening results and probes worry about what consequences it might have.

Based on this perceived ambivalence of a breast density notification, the women all described immediately feeling alarmed or panicking but to various degrees; some were very scared when they received the notification: *‘That's when I panicked: Oh my God, I've got cancer, I'm going to die…’* (Wendy, 54), while others were a bit worried: ‘*Although I didn't straight away jump on board and think: Oh my god, oh my god. I did worry about it’* (Lorraine, 65). The feelings of panic and worry can be considered an ambivalence or tension with the overall emphasis of empowerment by receiving breast density notifications.

### 3.3 Breast Density Notification Impacts Risk Perception

After the immediate worry, many started wondering if their breasts had suddenly become dense or if they had always been dense, which challenged their perceived risk of breast cancer.

*‘And then when I got the letter saying “your breasts are dense,” I thought: Well, have they all of a sudden become dense, or has this always been how it is? So, that felt a little bit weird, that if there was a problem before, why wasn't it said? Which made you feel a little bit—I don't know, anxious?…. Have I been having these things, thinking everything's all good when they're not really?’* (Carol, 65). Carol questioned if she had always had dense breasts and thus been at higher risk and expressed remorse that she had wrongfully thought she was at low risk. Carol continued: *‘Yeah, I probably feel like I've always been at higher risk now. That attitude, “it won't happen to me” kind of thing, when you get something in your face that says: Well, it might, because you're at a higher risk’* (Carol, 65). Carol had previously not thought of herself as at higher risk, but due to the information, she said she would discontinue the *‘it won't happen to me’* perception. In this way, the information about her breast density changed the way she perceived her personal risk and did now identify as at *‘higher risk’*.

Janet had previously considered her risk of breast cancer low due to other factors: *‘Yes. And I realise that I'm in the fortunate position of not having it in the family…. I'm aware that I'm not obese—if anything I'm underweight—I don't drink a lot of alcohol, I haven't got any other risk factors, so, that's good. But it's also made me not be complacent, which I probably was before. Now I've been told about the density thing, I think: Oh, okay’* (Janet, 62). After receiving the information about her breast density, Janet reinterpreted her personalised risk, which discredited her previous idea of being at low risk and described her previous perception as ignorant. In this way, the breast density notification led to increased perceived risk of breast cancer and discrediting of previous risk perceptions.

For other women, the information about breast density was used as a source of validation for existing perceptions about being at high risk. Jenny's mother died of breast cancer when she was in her forties: *‘…I know that there's a very good chance that at some point in my life, I'm going to get a positive result, or a negative result—a bad result; they're going to find something* (referring to breast cancer)*. So, knowing that because they're dense, I'm at a higher risk doesn't really mean too much to me personally’* (Jenny, 43). Because of Jenny's family history with breast cancer, she was already certain that she would get breast cancer, so receiving the breast density notification did not change the way she perceived her risk of breast cancer; rather, it just validated what she already knew. Similar ideas were shared by women with personal experiences with, for example, skin cancer and benign lumps previously detected in breast cancer screening. *‘So, I think I am at increased risk, because I've already been through something’* (Fang, 49).

Overall, the breast density notification affected women's perceptions of their individual risk and overall risk narratives, including considerations about previous experiences. The notifications were used to either verify women's existing high‐risk narratives or challenge previous low‐risk narratives about breast cancer.

### 3.4 Increased Awareness and Vigilance in Screening and Self‐Examinations

These risk considerations eventually led women to reevaluate their need to act and described how the notification had prompted a new kind of general awareness about the risk of breast cancer. This was described as being *‘more alert’* (Maria, 52), *‘self‐aware’* (Kate, 55), *‘keeping an eye on it’* (Alison, 69) or *‘have it on my mind a bit more’* (Kristen, 78).

The women described how this general awareness would play a role if they were to experience any aches: *‘I guess knowing now that if there's any kind of iffy‐ness, I would probably go sooner to have it checked out, rather than just think: “Yes, I've had my mammogram, it came back all right. All good for another 12 months.” I guess it's in the back of your mind now all the time to think: Well, maybe something didn't show up’* (Carol, 65). Here Carol refers to the lowered sensitivity of mammograms of dense breasts (masking), and that if she had any ‘*iffy‐ness*’ she would not rely on her mammograms but would go sooner to see the doctor. In this way, the awareness about having dense breasts for Carol changed her willingness and incentive to seek medical assistance.

Janet also said that if she had any concerns for her breast health, she would respond faster by going to see a doctor: ‘*So, if I ever have any concerns, I would definitely not leave it. I would go to the doctor. And if something came back that, “Oh, this is possibly something. Do you want us to look into it?” I'd say yes, because I'm aware that the mammogram may not pick up something that's there … rather than taking a chance, if you were to get any symptoms in the future, you would react to it quicker*’ (Janet, 62). Like Carol, Janet expressed increased readiness to seek medical assistance in case of concerns, but she also expressed an increased willingness to undergo potential diagnostic workup in future situations of uncertainty.

The general awareness about breast health and risk of breast cancer also influenced how women perceived the importance of breast cancer screenings, both in terms of intentions to attend and desire to attend more frequently. Linda noted: ‘*I will be very vigilant in following up every 2 years’* (Linda, 77). Notably, women used the word ‘*vigilant’* to describe their new awareness, especially in regard to mammograms: ‘*I definitely wouldn't miss another appointment. I would also make sure that I do the tests and stuff like that, I guess, yes, be vigilant with all that sort of thing and be mindful’* (Jan, 71). By receiving the breast density notification, the importance of breast cancer screening was reinforced, thus increasing the perceived need for screening vigilance.

The awareness also manifested in increased self‐examinations. Alison explained that she had done a few self‐examinations before but that she would intensify: ‘*Just in the shower, feeling for lumps now, probably more so than I would do, if I didn't know’* (Alison, 69). The vigilance in self‐examinations was echoed by Kristen: ‘*But just very aware that I've got a risk of breast cancer, so I just am very vigilant on self‐checks and that sort of thing. Not regularly, but quite often when I'm in the shower’* (Kristen, 78). Sharon explained that she felt an increased need to perform self‐examinations due to the lowered sensitivity of mammograms and thus lower reliance or confidence in the screenings alone: ‘*So, I gather that having density there means that it's a particular type of breast tissue … it's very hard for the mammogram to pick it up. So, I need to be very vigilant about myself breast‐checking for any lumps and things. So, it doesn't mean that it hasn't been picked up, but it means that if there's something there it's harder to pick up and it could be missed’* (Sharon, 69). Sharon thus motivates her increased vigilance for self‐examinations with the fear that cancer could be missed by mammograms.

## 4 Discussion

Women who reported feeling anxious after receiving their breast density notification generally experienced immediate but transient panic and heightened concern about their breast cancer risk. This combination of negative emotions and the information about being at increased risk challenged previous perceptions of breast cancer risk. As a result, women described an increased awareness of breast cancer, leading them to become more vigilant with self‐examinations and breast cancer screening. In this way, the breast density notification reinforced the importance of screening and raised awareness about breast cancer. This suggests that the notification prompted women to transform their personal narrative of risk, an understanding that shaped their past experiences and perceptions and influenced their future actions and health behaviours.

This lends insight into what it means to indicate anxiousness following a breast density notification. The immediate panic and worry, as well as increased awareness about breast cancer, are supported by earlier qualitative studies from the United States [10, 11, 13]. In these American studies, women considered breast density notification an indication that something was wrong or ‘not normal’ and were worried or anxious about their need for care [10, 11, 12, 13]. This interview study found that despite mammograms showing no signs of cancer, and women being told this upfront in their screening mammography result letter, women considered their test results ambivalent because of the threat of breast density and the masking effects of breast density impacting the sensitivity of mammograms.

Our analysis also points to an increased vigilance in breast health. On the one hand, being increasingly vigilant in health intuitively seems like a responsible and positive movement for women's health [29]. However, on the other hand, being more vigilant based on fear and worry about health introduced by a screening programme points to ethical tensions in health prevention. Being identified as at increased risk of a life‐threatening disease has previously been theorised as patient‐like identities, where people feel like they are sick with implications for psychological health [30, 31, 32, 33]. This indicates that being identified at risk of a disease might form patient‐like narratives, which in turn promote medicalisation and increase the perceived need for medical services [34, 35, 36]. This emphasises a tension between medicalisation and empowerment, which has previously been coined the ‘paradoxical value of health awareness’ [37].

### 4.1 Implications for Policy and Practice

In the RCT from which the women were sampled, participants received high‐quality information about breast density and the associated risk [9, 17]. First of all, this might be the reason women in this study expressed good understanding of the concepts, especially noteworthy compared to literature showing that women generally have poor understanding of breast density [8, 10, 11, 12, 13, 20, 38, 39, 40]. This might hint that attention to information material does help women understand what breast density is and why it is important. Second, this presents a best‐case scenario, in which high‐quality information could alleviate potential psychosocial impacts. However, this qualitative study found that women still experienced immediate panic and worry, driving increased attention to breast health, including increased self‐exams and increased desire for breast cancer screening. Increased vigilance and empowerment in health may present positive advances for women's health; however, the current evidence for increased screening participation and self‐exams is most likely small to none [41, 42]. Downstream, this may have implications for individuals, health systems, and the environment [16, 43, 44, 45]. Policy makers and screening services should consider this tension when deciding to implement breast density notifications.

### 4.2 Strengths and Limitations

Telephone interviews have several limitations as it is more challenging to establish rapport and allow for genuine disclosure, particularly when discussing psychosocial aspects. Therefore, we asked third‐person questions as a form of vignette‐like design, where we used hypothetical prompts to elicit thinking around breast density notifications and help participants articulate experience or reasoning that might not emerge spontaneously [22]. We were also conscious about this when performing the analysis to not overinterpret without sufficient information about contextual factors [22]. A strength of this study is the contribution to the sparse evidence on the psychosocial mechanisms and impacts of breast density notification, including health‐care behaviour and needed support.

## 5 Conclusions

In this study, women who indicated anxiousness following their mammography results letter and notification of dense breasts experienced negative psychosocial consequences, including immediate panic, worry about breast cancer and changes to risk narrative, including perceived personal risk and need for action. This points to a tension between empowerment and derivative worry, which should be considered in current notification practices as well as decisions on implementation of breast density notification.

## Author Contributions

**Emma Grundtvig Gram:** conceptualisation, data curation, formal analysis, funding acquisition, methodology, project administration, writing – original draft, writing – review and editing. **Claire Hudson:** data curation, formal analysis, project administration, validation, writing – review and editing. **Nehmat Houssami:** conceptualisation, funding acquisition, writing – review and editing. **Kirsten McCaffery:** conceptualisation, writing – review and editing. **Jennifer Isautier:** data curation, formal analysis, project administration, writing – review and editing. **Brooke Nickel:** conceptualisation, data curation, formal analysis, funding acquisition, methodology, project administration, supervision, validation, writing – review and editing.

## Ethics Statement

Ethics approval has been obtained at the Gold Coast Hospital and Health Service Ethics Committee (HREC/2023/QGC/89770).

## Consent

Informed consent forms were sent to the women through email, including information about the study. Participants were informed that they could withdraw their consent at any time during the study and were given contact details if they had subsequent worries or questions they would like to discuss.

## Conflicts of Interest

E.G., B.N. and K.M. are members of the Preventing Overdiagnosis Scientific Committee.

## Data Availability

The authors have nothing to report.

## Appendix 1 ‐ Interview Guide

**Introduction**

Thank them for their participation

Inform them about the purpose of the study and confidentiality measures

Obtain an informed choice

Ask them to tell a bit about themselves: age, interests, hobbies and so forth

**Impact of breast density notification**

Ask them to reproduce their experience with receiving breast density notification

Ask them about their feelings upon receiving the notification of high breast density

Ask them if and how it has impacted well‐being and daily life

Ask them if and how it has impacted thoughts about the body and breasts

**Coping mechanisms/Healthcare interactions**

Ask them about their personal pathway after receiving the breast density notification

Ask them to describe your thoughts about seeking support after the notification

**General discussion about breast density notifications**

Ask them about their general thoughts on breast density notifications

**Closing**

Thank them for their time and insights

Offer additional support or resources if needed.

Provide contact information for further questions or support.
